# Genome Sequences of 12 Spore-Forming *Bacillus* Species, Comprising *Bacillus coagulans, Bacillus licheniformis, Bacillus amyloliquefaciens, Bacillus sporothermodurans*, and *Bacillus vallismortis*, Isolated from Foods

**DOI:** 10.1128/genomeA.00103-16

**Published:** 2016-05-12

**Authors:** Antonina O. Krawczyk, Anne de Jong, Siger Holsappel, Robyn T. Eijlander, Auke van Heel, Erwin M. Berendsen, Marjon H. J. Wells-Bennik, Oscar P. Kuipers

**Affiliations:** a Molecular Genetics, University of Groningen, Groningen, The Netherlands; b NIZO food research, Ede, The Netherlands; c Top Institute Food and Nutrition (TIFN), Wageningen, The Netherlands

## Abstract

Here, we report the draft genomes of twelve isolates of five different *Bacillus* species, all spore-forming, Gram-positive bacteria.

## GENOME ANNOUNCEMENT

*Bacillus* species have the ability to form endospores (spores). *Bacillus* spores are ubiquitously present in soil, and transmission to food products can take place (1). Spores are highly resistant to environmental stresses and can food processing conditions. Germination of spores followed by growth may result in food spoilage (2). Here, were report the draft genome sequences of twelve stains belonging to five different *Bacillus species* that were isolated from foods: Four strains of *Bacillus coagulans*, four strains of *Bacillus licheniformis*, two strains of *Bacillus amyloliquefaciens*, one strain of *Bacillus sporothermodurans*, and one strain of *Bacillus vallismortis*. Comparison of the sequenced genomes with those of *B. subtilis* may provide insight into variations in the sporulation and germination processes (3). Furthermore, genome mining can provide insight into the genomic potential of strains in relation to predicted phenotypic traits and their ability to produce toxins involved in food poisoning, such as lichenysin in *B. licheniformis* (4).

Twelve strains of different isolation sources (Table 1), were grown overnight in 10 ml of brain heart infusion (BHI) broth (Difco) at 37°C. The overnight cultures were diluted 100-fold in fresh medium and incubated at 37°C until the culture reached an optical density (at 660 nm) of approximately 0.5, and cells were then harvested by centrifugation at 5000 rcf. DNA was isolated as described previously (5). The isolated DNA was sheared to 500-bp fragments in a Covaris (KBioscience) ultrasone device for preparing the Next-Generation Sequencing (NGS) library preps using the paired-end NEB NExtGen library preparation kit. The prepared libraries were 101 bases paired-end sequenced on an Illumina HiSeq2000 by multiplexing 12 samples per flow cell. *De novo* paired-end assembly of the genomes was performed using Velvet (6). The genomes were annotated using RAST (7), and scaffolds were mapped on the closest neighbor according to RAST using CONTIGuator (8). Protein annotations were extended using Interproscan (9) and BAGEL3 (10) was used for identification of putative bacteriocin gene clusters.

**TABLE 1:** Genome features and GenBank accession numbers of the strains

Strain	Species	Source of isolation	Bioproject no.	Accession no.
B4098	*Bacillus coagulans*	Chinese tomato	PRJNA270593	LQYG00000000
B4100	*Bacillus coagulans*	Low pH sauce	PRJNA270593	LQYH00000000
B4099	*Bacillus coagulans*	Indian curry	PRJNA270593	LQYI00000000
B4096	*Bacillus coagulans*	Tomato supreme	PRJNA270593	LQYJ00000000
B4092	*Bacillus licheniformis*	Buttermilk powder	PRJNA270588	LQYK00000000
B4090	*Bacillus licheniformis*	Pea soup	PRJNA270588	LQYL00000000
B4091	*Bacillus licheniformis*	Mushroom soup	PRJNA270588	LQYM00000000
B4102	*Bacillus sporothermodurans*	Indian curry	PRJNA270602	LQYN00000000
B4140	*Bacillus amyloliquefaciens*	Pizza	PRJNA270600	LQYO00000000
B425	*Bacillus amyloliquefaciens*	Sterilized milk	PRJNA270600	LQYP00000000
B4164	*Bacillus licheniformis*	Unknown food	PRJNA270588	LQYQ00000000
B4144	*Bacillus vallismortis*	Quiche	PRJNA270602	LQYR00000000

### Nucleotide sequence accession numbers.

The genome sequences of the twelve *Bacillus* sp. strains have been deposited as whole-genome shotgun projects at DDBJ/EMBL/GenBank under the accession numbers listed in Table 1.
